# Microvessel density and VEGF expression are prognostic factors in colorectal cancer. Meta-analysis of the literature

**DOI:** 10.1038/sj.bjc.6603176

**Published:** 2006-06-13

**Authors:** G Des Guetz, B Uzzan, P Nicolas, M Cucherat, J-F Morere, R Benamouzig, J-L Breau, G-Y Perret

**Affiliations:** 1 APHP. Department of Oncology, Hôpital Avicenne, 125 route de Stalingrad, Bobigny 93009, France; 2 Department of Pharmacology, Hôpital Avicenne, 125 route de Stalingrad, Bobigny 93009, France; 3 Department of Clinical Pharmacology, EA643, Lyon-1 University, Laennec Faculty of Medicine, rue G. Paradin, Lyons 69008, France; 4 Department of Gastroenterology, Hôpital Avicenne, 125 route de Stalingrad, Bobigny 93009, France

**Keywords:** colorectal cancer, microvessel density, VEGF expression, prognostic factors, survival

## Abstract

We performed a meta-analysis of all published studies relating intratumoural microvessel density (MVD) (45 studies) or vascular endothelial growth factor (VEGF) expression (27 studies), both reflecting angiogenesis, to relapse free (RFS) and overall survival (OS) in colorectal cancer (CRC). For each study, MVD impact was measured by risk ratio between the two survival distributions with median MVD as cutoff. Eleven studies did not mention survival data or fit inclusion criteria, six were multiple publications of same series, leaving 32 independent studies for MVD (3496 patients) and 18 for VEGF (2050 patients). Microvessel density was assessed by immunohistochemistry, using antibodies against factor VIII (16 studies), CD31 (10 studies) or CD34 (seven studies). Vascular endothelial growth factor expression was mostly assessed by immunohistochemistry. Statistics were performed for MVD in 22 studies (the others lacking survival statistics) including nine studies (*n*=957) for RFS and 18 for OS (*n*=2383) and for VEGF in 17 studies, including nine studies for RFS (*n*=1064) and 10 for OS (*n*=1301). High MVD significantly predicted poor RFS (RR=2.32 95% CI: 1.39–3.90; *P*<0.001) and OS (RR=1.44; 95% CI: 1.08–1.92; *P*=0.01). Using CD31 or CD34, MVD was inversely related to survival, whereas it was not using factor VIII. Vascular endothelial growth factor expression significantly predicted poor RFS (RR=2.84; 95% CI: 1.95–4.16) and OS (RR=1.65; 95% CI: 1.27–2.14). To strengthen our findings, future prospective studies should explore the relation between MVD or VEGF expression and survival or response to therapy (e.g. antiangiogenic therapy). Assessment of these angiogenic markers should be better standardised in future studies.

Colorectal cancer (CRC) is the third most common cancer and the fourth most frequent cause of cancer deaths worldwide (Weitz *et al*, 2005). The main prognostic factors in CRC are lymph node involvement, size of the tumour and local diffusion of disease (Hellman and Rosenberg, 2001; Hermanek and Sobin, 1995). However, these prognostic factors do not fully predict individual clinical outcome especially among stage II and III patients. Therefore, to improve clinical care, biological prognostic markers must be identified, especially for localised tumours.

Angiogenesis consists in the formation of new blood vessels from the endothelium of the existing vasculature. When a new tumour reaches the size of 1–2 mm, its ulterior growth requires the induction of new blood vessels, which may lead to the development of metastases. Angiogenesis is dependent on the balance between many stimulatory and inhibitory factors. Proangiogenic factors, such as vascular endothelial growth factor (VEGF), bind to sites on endothelial cells that lead to their proliferation.

Concerning the relationship between angiogenesis and clinical outcome, CRC has been one of the most studied tumours after breast cancer (Uzzan *et al*, 2004). Microvessel density (MVD), as a surrogate marker of tumoral angiogenesis, has been proposed to identify patients at high risk of recurrence. Microvessel density assessment is the most commonly used technique to quantify intratumoral angiogenesis in cancer. It was first developed by Weidner *et al* (1991) in 1991 and used panendothelial immunohistochemical staining of blood microvessels, mainly with Factor VIII related antigen (F. VIII Ag or von Willebrand's factor), CD31 or CD34, rarely CD105. Some authors used Chalkley count or computerised image analysis systems, both aimed to minimise the subjectivity in the quantification of MVD (Chalkley, 1943).

Several methods were employed for the assessment of VEGF expression in the tumours: most often immunohistochemistry, but also RT–PCR or Northern Blot. Circulating VEGF may be related to the tumour, but is also certainly produced by platelets, granulocytes, monocytes; in addition, its determination may be technically difficult (Vermeulen *et al*, 2002). Therefore, we decided not to include the studies relating circulating VEGF to survival in our meta-analysis (MA).

Many observational retrospective studies have concluded that MVD is inversely related to survival in CRC, but other studies did not reach this conclusion (Poon *et al*, 2003). To determine whether angiogenesis, assessed by its surrogate end point MVD, and by the expression of the main angiogenic factor VEGF, is prognostic factor in CRC, we undertook a systematic review of the literature with a meta-analysis. Meta-analyses of observational studies may provide a useful tool for understanding and quantifying sources of variability in results across studies (Stroup *et al*, 2000).

The aim of our study was to test the hypothesis that initially assessed MVD or VEGF expression would predict overall survival (OS colon cancer-related death) and/or relapse-free survival (RFS, recurrence at any site) in the global population of operated colon cancer patients. By doing so, we tried to contribute to convert MVD and/or VEGF expression from candidate to accepted prognostic factors in CRC. Actually, we performed four major meta-analyses including studies dealing with either MVD or VEGF expression for both OS and RFS. We also tried to study the relationship between MVD or VEGF expression and survival across various stages of CRC. Finally, we were interested to determine which of the two markers might be considered as the best angiogenic prognostic factor in localised or metastatic disease.

## MATERIALS AND METHODS

### Publication selection

We performed our meta-analysis according to a predefined written protocol. To be eligible, studies had to deal with colon or rectum cancer, whatever the stage at inclusion of patients in the individual studies, and to assess the relationship between MVD or VEGF expression on one side and RFS or OS on the other side. Studies (full articles) were identified by an electronic search using online PubMed, with two distinct sets of key words used simultaneously in each set, namely ‘colorectal cancer, neovascularization, prognosis’ and ‘colorectal cancer, VEGF expression, prognosis’. Last query was updated on 7 October 2004. We did another electronic search with the same key words using online EMBASE, which was unable to retrieve additional pertinent references. Our initial selection of articles relied on careful reading of their abstracts. Abstracts were reviewed from ASCO proceedings of the annual meetings from 1998 to 2004, but no additional data were found. We also screened references from the relevant literature, including all of the identified studies, but also reviews and editorials (Papamichael, 2001; Poon *et al*, 2003). We wrote or e-mailed to the authors of 20 studies (see Appendix A) for additional information and, in 5 occasions obtained the data needed for the meta-analytic calculations (Choi *et al*, 1998; Ishikawa *et al*, 1999; Harada *et al*, 2001; Khorana *et al*, 2003; Galizia *et al*, 2004). We tried carefully to avoid duplication of data, by examining for each publication the names of all authors and the different medical centres involved. We excluded studies when their recruitment came from two distinct retrospective cohorts with different survivals (Banner *et al*, 1998; Nanashima *et al*, 1998; Barozzi *et al*, 2002; Saad *et al*, 2004), because we deemed their results could be biased.

### Methodological assessment

Information was carefully extracted from all full publications in duplicate by the two readers (Gaëtan Des Guetz and Bernard Uzzan), using a standardised data collection form, including the following items: complete reference of the publication, original publication or update of a former publication, mode of making up of the series of cases, median duration of follow-up, number of patients included in the study, mean or median age, sex, anticancer treatment(s) during follow-up, histological type (adenocarcinoma or mucinous), tumour size, stage of disease, grade (good, moderate or poor differentiation), nodal status, optical reading of the slides with or without Chalkley count or image analysis system, number of readers of the slides, blinded reading (reader of the slides unaware of clinical information), type(s) of immunohistochemical staining, number of hot spots examined, magnification used, area of the field read, cutoff value for MVD (median MVD, unless otherwise stated, for example optimal cu-off (Takebayashi *et al*, 1996; Galindo Gallego *et al*, 2000), semiquantitative intensity of the VEGF expression (0, +, ++ or +++), number of events in each category of MVD or VEGF, RFS or OS or both, and results of uni- and multivariate analyses. Chalkley count was used in two studies (White *et al*, 2002; Li *et al*, 2003). Disagreements were resolved by consensus between the two readers. In case of persistent disagreement, the final decision was made by our experts (Gérard Perret for clinical evaluation, and Michel Cucherat for methodological and statistical assessment of data). We did not set a predefined minimal number of patients for a study to be included in our meta-analysis, nor a minimal duration of median follow-up. We did not weigh each study by a quality score, because no such score has received general agreement for use in a meta-analysis, especially of observational studies, making more difficult the evaluation of its usefulness (Altman, 2001). Studies were not blinded to our readers, but exclusions were always decided without knowledge of the global result of each study. When duplicate studies were retrieved, we included in our systematic review, the study involving the highest number of patients from which data could be extracted (usually the latest). This was done to avoid overlapping between cohorts. Studies were usually retrospective, but sometimes consisted in a cohort of consecutive patients. Although their methodological quality and the reliability of their conclusions were variable, their design was almost similar, a favourable condition for our meta-analysis.

### Statistical methods

In each study, the relationship between MVD or VEGF expression and survival was considered significant when the *P*-value for the statistical test comparing survival distributions between the groups with high and low MVD (usually with median MVD as cutoff) was inferior to 0.05 in univariate analysis (two-tailed test). A study was termed ‘positive’ or conclusive when a high MVD predicted poorer survival and ‘negative’ or inconclusive when a high MVD did not predict a poor survival. In a few studies, a high MVD even predicted better survival (Lindmark *et al*, 1996; Abdalla *et al*, 1999; Prall *et al*, 2003). Whenever possible, the parameter MVD was considered as a binary outcome and dichotomised by using its observed median. For the quantitative aggregation of survival results, we measured the impact of MVD on survival by estimating the risk ratio (RR) between the high or low MVD groups. For each trial, this RR was estimated by a method depending on the data provided in the publication. The simplest method consisted in the direct collection of RR, hazard ratio, or odds ratio, and their 95% confidence interval (CI) from the original article (Amaya *et al*, 1997; Tanigawa *et al*, 1997; Ishigami *et al*, 1998; Vermeulen *et al*, 1999; Maeda *et al*, 2000; White *et al*, 2002; Kaio *et al*, 2003b; Zheng *et al*, 2003; Galizia *et al*, 2004; Liang *et al*, 2004; Tamura *et al*, 2004). If not available, we looked at the total numbers of events and the numbers of patients at risk in each group to determine the RR estimate. When data were only available as graphical survival plots, the calculations were carried out only if the number of steps on the curves equalled the number of events given in the publication, assuming that the rate of censored patients was constant during the study follow-up (Parmar *et al*, 1998). In two studies, MVD was expressed as a continuous variable with no possibility to convert the corresponding HRs to their dichotomous counterparts so that they could not be incorporated into our meta-analytic calculations (Takebayashi *et al*, 1996; Lackner *et al*, 2004).

The heterogeneity between studies being difficult to investigate reliably, we chose to incorporate the assumption that the effect on survival between studies was not identical but followed some unknown distribution. Thus, we calculated a pooled random RR estimate and its 95% CI by using a random-effect model (Der Simonian and Laird's method). This method is more conservative because the CI around the random RR pooled estimate is wider than the CI around the fixed RR pooled estimate. By convention, an observed RR **>**1 implied a worse prognosis in the high MVD or VEGF expression group. The detrimental impact of angiogenesis on survival was deemed statistically significant whenever the lower of the 95% CI of the overall RR was **>**1. Comparisons of proportions of studies with or without various characteristics were made by *χ*^2^ tests. The statistical calculations for our meta-analyses were performed with EasyMA.net, Internet distributed application (Department of Clinical Pharmacology, Cardiology Hospital, Lyons, France) (Cucherat *et al*, 1997).

## RESULTS

**Figure 1:**
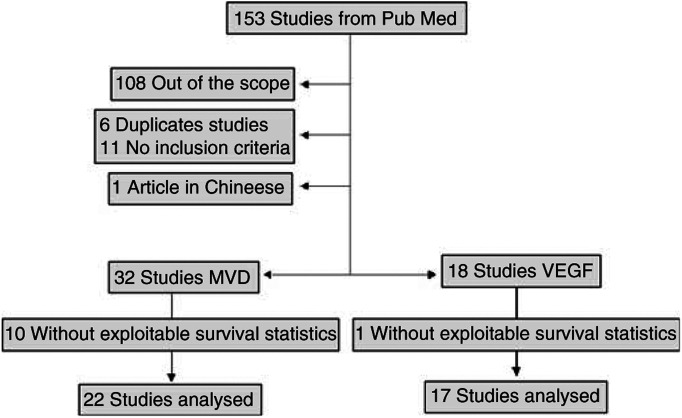
Flow chart of the meta-analysis.

Our electronic data search using online PubMed and EMBASE retrieved a total of 153 references (107 dealing with MVD and 46 dealing with VEGF expression; full list available on request) including duplicate references since some publications studied both markers. After exclusion of the references which were out of the scope of our meta-analysis, there remained 45 studies dealing with MVD (see Appendix B) and 27 dealing with VEGF expression (see Appendix C), representing a total of 56 independent studies. Some of these articles did not fulfil our inclusion criteria (mainly because they did not mention survival data), six for MVD (Vermeulen *et al*, 1995; Banner *et al*, 1998; Nanashima *et al*, 1998; Kondo *et al*, 2000; Barozzi *et al*, 2002; Saad *et al*, 2004) and five for VEGF (Nanashima *et al*, 1998; Kondo *et al*, 2000; Seto *et al*, 2000; Barozzi *et al*, 2002; Saad *et al*, 2004). One study was written in Chinese language, with an English abstract and did not seem to mention survival data (Liu *et al*, 1999). Some publications corresponded to duplicate studies of the same marker, six for MVD (Amaya *et al*, 1997; Abdalla *et al*, 1999; Galindo-Gallego *et al*, 2000; Furudoi *et al*, 2002; Kaio *et al*, 2003b; Onogawa *et al*, 2004) and four for VEGF expression (Amaya *et al*, 1997; Furudoi *et al*, 2002; Kaio *et al*, 2003b; Onogawa *et al*, 2004) (Figure 1).

Almost all studies (*n*=40) used Dukes staging or derived classification (Astler-Coller). Two studies used only TNM staging (Tanigawa *et al*, 1997; Fox *et al*, 1998). Several studies used both classifications (see Appendix D). To better describe the patients included in our meta-analysis, we used Dukes staging whenever possible. For one study (Ishikawa *et al*, 1999), in the absence of lymph node involvement or metastasis, we could convert stage T_1–3_N_0_M_0_ into stage A or B.

The main features of the eligible studies for MVD are summarized in Table 1. Thirty-two independent studies representing 3496 patients with mean age of 64.7 years (1740 male patients, 1400 female patients) included 1449 colon and 673 rectum cancers. These studies included 286 stage A, 1315 stage B, 1085 stage C and 388 stage D. There were only eight series of consecutive patients (Lindmark *et al*, 1996; Ishikawa *et al*, 1999; Vermeulen *et al*, 1999; Pietra *et al*, 2000; Prall *et al*, 2003; Shan *et al*, 2003; Galizia *et al*, 2004; Liang *et al*, 2004) and one prospective study (Nanni *et al*, 2002), compared to 23 retrospective studies.

Finally, statistical calculations could be performed in 22 studies for MVD including nine studies (*n*=957) for RFS (Engel *et al*, 1996; Takahashi *et al*, 1997; Choi *et al*, 1998; Ishikawa *et al*, 1999; Galindo Gallego *et al*, 2000; Nanni *et al*, 2002; Shan *et al*, 2003; Galizia *et al*, 2004; Liang *et al*, 2004) and 18 for OS (*n*=2383) (see Appendix E). High MVD significantly predicted poor RFS (RR=2.32; 95% CI: 1.39–3.90; *P*<0.001) and poor OS (RR=1.44; 95% CI: 1.08–1.92; *P*=0.01).

**Figure 2:**
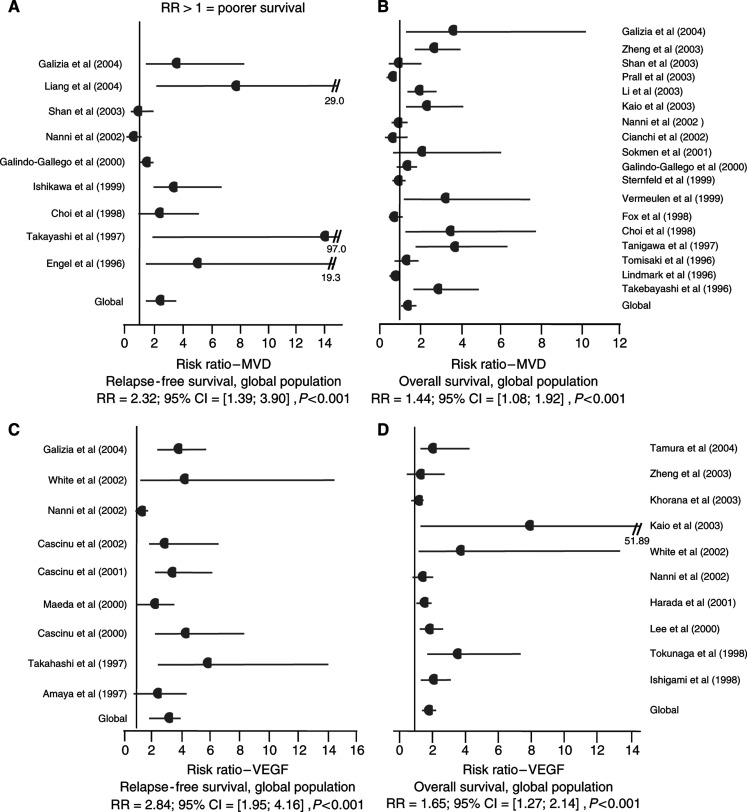
Results of the four meta-analyses (**A**–**D**). RRs estimated with DerSimonian and Laird's random model.

Meta-analysis was also performed to relate VEGF expression and survival across all exploitable studies. The main features of eligible studies for VEGF are summarized in Table 2. Eighteen independent studies with 2050 patients with mean age 63.3 years (1041 male patients, 750 female patients) included 1104 colon cancers and 202 rectum cancers. These studies included 130 stage A, 472 stage B, 626 stage C and 149 stage D. Statistical calculations were performed for VEGF in 17 studies, including nine studies (*n*=1064) (Amaya *et al*, 1997; Takahashi *et al*, 1997; Cascinu *et al*, 2000; Maeda *et al*, 2000; Cascinu *et al*, 2001; Cascinu *et al*, 2002; Nanni *et al*, 2002; White *et al*, 2002; Galizia *et al*, 2004) for RFS and 10 for OS (*n*=1301) (Ishigami *et al*, 1998; Tokunaga *et al*, 1998; Lee *et al*, 2000; Harada *et al*, 2001; Nanni *et al*, 2002; White *et al*, 2002; Kaio *et al*, 2003b; Khorana *et al*, 2003; Zheng *et al*, 2003; Tamura *et al*, 2004). High VEGF significantly predicted poor RFS (RR=2.84; 95% CI: 1.95–4.16; *P*<0.001) and poor OS (RR=1.65; 95% CI: 1.27–2.14; *P*<0.001). All four major meta-analyses gave statistically significant results, favouring a link between high MVD and VEGF expression and poor survival (see Figure 2).

We have shown previously in our meta-analysis relating MVD to survival in breast cancer that CD 31 and CD 34 were the best markers to predict survival compared with factor VIII (Uzzan *et al*, 2004). Our present work confirms these findings for CRC. Actually a specific meta-analysis performed by using CD 31/CD34 in CRC gave higher RRs than the global meta-analysis (data not shown).

To determine whether MVD and/or VEGF expression are prognostic factors more suited to limited (stage A/B) or advanced disease (stage C/D), we divided the studies into those including a majority of limited forms, those including a majority of advanced forms and those where limited and advanced forms were balanced (mixed studies) which we omitted for being too few and ill-defined. For RFS and MVD, the RR for the studies with advanced forms (*n*=3) was higher than the RR for the studies with limited forms (*n*=7) (3.23 *vs* 2.49, these two^**^ RRs being significantly different from 1 but not different from each other). These results are in favour of a prognostic role of MVD either in local or in advanced disease. For VEGF and RFS studies with localised disease (*n*=4), we found a RR of 4.05 (*P*<0.001) compared with a RR of 3.41 (*P*<0.001) for the studies with advanced disease (*n*=2). For MVD and VEGF expression and for OS, the RRs of the studies with limited and advanced disease were also significantly >1, but less significant than for RFS.

Finally, we compared the ability of both angiogenic markers to predict survival by calculating the ratio of their RRs; for RFS, this ratio RR VEGF/RR MVD was found to be 1.22 (95% CI: 0.50–2.98), not significantly different from 1. However, the 95% CI width was smaller for VEGF than for MVD. The results were similar for OS.

## DISCUSSION

Our overview and meta-analysis of all published studies from which statistical data could be obtained or calculated showed that high MVD and VEGF expression, markers of angiogenesis, did indeed predict poor survival in patients with CRC.

However, our conclusions should be tempered for several reasons. First, the overall link we elicited between MVD and survival, although statistically significant, was rather weak, with a global RR of 1.44 for OS and 2.32 for RFS. However, for VEGF expression, these links were stronger (1.65 for OS; 2.84 for RFS). Empirically, RRs >2 are considered strongly predictive (Hayes *et al*, 2001). Both markers appeared more predictive for RFS than for OS, not surprisingly since OS is a more stringent parameter than RFS, harder to be influenced by treatments. We performed the meta-analyses including selectively the studies involving consecutive patients, supposed to be of better methodological quality, and found, rather unexpectedly, that the relation between survival and markers of angiogenesis was not improved for RFS, and even deteriorated for OS.

Our meta-analysis has several limitations. First, the level of evidence provided by MA of retrospective observational studies is lower than that of randomised controlled trials. Also, it relied on publications and not on individual data. But a meta-analysis on individual data would require the implication of many pathologists and a time-consuming processing of materials because of the large number of patients included in the studies and especially of their rather poor quality. There were several potential sources of heterogeneity between studies, but the Der Simonian and Laird method we used (random effect model) took them into account.

Studies may have differed in the baseline characteristics of patients included (age, tumour size, and stage), the adjuvant treatment they might have received for their cancer, the number of patients, the duration of follow-up. We attempted to minimise publication bias by making our literature search as complete as possible, using two databases (PubMed and EMBASE), reviewing ASCO meetings proceedings from 1998 to 2004 and crosschecking references. The discrepancies in the conclusions of various published studies could have encouraged researchers to publish their data whatever their results, thus limiting such publication bias. All publications but one (Liu *et al*, 1999) were written in English language. The immunohistochemical marker used to assess MVD, or the method of microvessel count itself were sources of variability and represented potential selection biases. Weidner *et al* (1991) used an antibody against factor VIII-related antigen, staining mainly mature vessels and cross-reacting with lymphatic endothelium. This marker remained the most used in the studies we reviewed. Several recent studies used antibodies directed against CD31 or CD34, best prognostic markers in CRC.

Many variations to the method of MVD assessment exist, although most studies used a technique similar to that of Weidner *et al* (1991). The size of the area examined varied between studies. Some authors considered the mean or the highest value among three or more determinations of MVD at different fields of the same hot spot (Saclarides *et al*, 1994; Lindmark *et al*, 1996; Tanigawa *et al*, 1997; van Triest *et al*, 2000). Some measured MVD as the mean or highest value at several hot spots (Sokmen *et al*, 2001; Shan *et al*, 2003; Galizia *et al*, 2004; Lackner *et al*, 2004). The choice of the cutoff value for MVD varied among studies, many used median MVD. In future studies, the assessment of these angiogenic prognostic factors should be better standardised, especially for patients for whom adjuvant therapy is recommended (Vermeulen *et al*, 2002).

Prognostic biomarkers may be useful for identifying high-risk patients, leading to an improvement in their clinical or therapeutic management (Weitz *et al*, 2005). Whereas in stage III CRC patients, adjuvant chemotherapy has been consistently shown to increase OS, in stage II it provides a small benefit, still uncertain. Meta-analyses gave conflicting results, one concluding to a small beneficial effect of chemotherapy (Mamounas *et al*, 1999) and the other to the absence of benefit (Gill *et al*, 2004). No study analysed separately the prognostic role of angiogenic markers among colon or rectum cancers. Microvessel density or VEGF expression might be predictive factors of the response to anti-angiogenic drugs (bevacizumab), now in phase III or IV trials (Hurwitz *et al*, 2004). In metastatic CRC, MVD and VEGF expression did not predict the favourable response to bevacizumab in one retrospective study derived from the pivotal efficacy trial (Jubb *et al*, 2006). Conversely, VEGF predicted rectal tumour response to preoperative radiotherapy (Zlobec *et al*, 2005). Therefore, pathological markers such as MVD or VEGF expression would be helpful for individualisation of patients who would benefit from anti-angiogenic therapy.

We found a trend to a relationship between tumour stage (limited, advanced) and the capacity of angiogenesis markers to predict survival. There are pathophysiological grounds for such a relationship, since angiogenesis is a very early phenomenon in colon carcinogenesis and it is also essential to metastasis (Garcea *et al*, 2004; Wali *et al*, 2005). However, our findings might also be artefactual, since the definition of the three categories of studies was imprecise and there were few studies. According to our results, VEGF seemed to be a rather better angiogenic predictor of survival than MVD, due to a narrower 95% CI although the ratio of their RRs was not significantly different from 1. These last results should be interpreted cautiously, since this double factor analysis would ideally be performed on individual patients data.

The following recommendations should be made to future authors: include a large series of consecutive patients from a single cohort, stratify by tumour stage, fully describe the clinical characteristics of the study population, use antibodies directed against either CD31 or CD34 for immunostaining, present the results both as comparison of survival curves and as multivariate regression analysis and provide a full description of survival events to allow calculations. Future studies should include more homogeneous populations and should be prospective.

To conclude, our meta-analysis, representing a quantified synthesis of all published studies, found a statistically significant inverse relationship between angiogenesis, assessed by MVD or VEGF expression, and survival, confirming that, like breast cancer, human invasive colorectal cancer is an angiogenesis-dependent malignancy.

### Addendum

Our PubMed query was ultimately updated to 14 February 142006. The relation between survival and MVD was assessed in only two additional articles, a positive study including 60 patients for RFS (Acikalin *et al* (2005) Tumour angiogenesis and mast cell density in the prognostic assessment of colorectal carcinomas. *Dig Liver Dis* **37:** 162–169) and a study of borderline significance including 92 patients for OS (Yonenaga *et al* (2005) Absence of smooth muscle actin-positive pericyte coverage of tumor vessels correlates with hematogenous metastasis and prognosis of colorectal cancer patients. *Oncology* **69:** 159–166). The relation between survival and VEGF expression was assessed in two other articles, one negative study including 109 stage II colon cancers assessed for OS (Ochs *et al* (2004) Expression of vascular endothelial growth factor and HER2/neu in stage II colon cancer and correlation with survival. *Clin Colorectal Cancer* **4:** 262–267) and one positive study including 69 patients assessed for RFS and OS (Ferroni *et al* (2005) Prognostic value of vascular endothelial growth factor tumor tissue content of colorectal cancer. *Oncology* **69:** 145–153). After incorporation into our meta-analysis of these four additional studies, the global RRs were very similar to the old ones, which could be expected from the small numbers of patients added and consequently the large CIs surrounding the RRs of these new studies. For MVD, the new RRs were 2.43 (95% CI: 1.49–3.96) for RFS and 1.46 (95% CI: 1.10–1.92) for OS. For VEGF, the new RR was 2.92 (95% CI: 2.04–4.17) for RFS (the RR for OS did not change. Thus, the conclusions of our four meta-analyses are identical before and after incorporation of these four new studies.

## Figures and Tables

**Table 1:** Main characteristics of the studies relating microvessel density to MVD survival

**First author Year of issue (reference)**	**Study from PubMed**	**Study design**	***N* (M/F)**	**Colon (*n*)**	**Rectum (*n*)**	**Blinded reading**	**Reader(s) (*n*)**	**Mode of reading**	**Antibody**	**Extension**	**RR estimate**	**Survival analysis**	**Results**
Galizia *et al* (2004)	Yes	C	104 (73/31)	104	0	Yes	2	Optical	CD34	Limited	Given by authors	OS, RFS	Negative
Lackner *et al* (2004)	Yes	R	70 (39/31)	49	21	?	?	Optical	FVIII CD34	Mixed	Missing	?	Positive
Liang *et al* (2004)	No	C	114 (60/54)	77	37	Yes	1	Optical	CD34	Advanced	Reported in text	RFS	Positive
Kaio *et al* (2003a, 2003b)	Yes	R	152 (94/58)	?	?	?	?	Optical	CD34	Mixed	Survival curves	OS	Negative
Prall *et al* (2003)	Yes	C	173 (87/86)	?	?	?	1	Optical	FVIII	Mixed	Survival curves	OS	Inverse
Li *et al* (2003)	Yes	R	111 (65/46)	83	28	?	?	Chalkley	CD105 CD34	Mixed	Survival curves	OS	Positive
Shan *et al* (2003)	Yes	C	104 (55/49)	72	32	Yes	2	Optical	FVIII	Limited	Data extrapolated	OS, RFS	Negative
Zheng *et al* (2003)	Yes	R	97 (58/39)	?	?	?	?	Optical	CD34	Mixed	Reported in text	OS	Positive
Cianchi *et al* (2002)	Yes	R	84 (60/24)	0	84	Yes	2	Optical	CD31	Limited	Data extrapolated	OS	Negative
Nanni *et al* (2002)	No	P	263 (137/126)	263	0	Yes	2	Optical	FVIII	Mixed	Data extrapolated	OS, RFS	Negative
White *et al* (2002)	Yes	R	84 (42/42)	62	22	Yes	2	Chalkley	CD31 FVIII	Limited	Missing	OS, RFS	Inverse
Sokmen *et al* (2001)	Yes	R	29 (18/11)	0	29	Yes	2	Automated	FVIII	Mixed	Survival curves	OS	Positive
Galindo-Gallego *et al* (2000)	Yes	R	126 (70/56)	87	39	?	?	Optical	CD34	Limited	Survival curves	OS, RFS	Negative
Pietra *et al* (2000)	Yes	C	119 (62/57)	78	41	Yes	2	Optical	CD31	Mixed	Missing	OS, RFS	Negative
Van Triest *et al* (2000)	Yes	R	32 (13/19)	26	6	Yes	2	Optical	CD31	Mixed	Missing	Missing	ND
Giatromanolaki *et al* (2002)	No	R	106 (65/41)	77	29	?	?	Optical	CD31	Mixed	Missing	OS	Positive
Ishikawa *et al* (1999)	Yes	C	57 (34/23)	0	57	Yes	2	Optical	CD31	Limited	Survival curves	RFS	Positive
Sternfeld *et al* (1999)	Yes	R	146 (?)	70	76	?	1	Optical	CD31	Limited	Survival curves	OS	Positive
Vermeulen *et al* (1999)	No	C	145 (75/70)	109	36	?	?	Optical	CD31	Advanced	Reported in text	RFS	Positive
Choi *et al* (1998)	Yes	R	127 (72/55)	?	?	Yes	2	Optical	FVIII	Advanced	Survival curves	OS, RFS	Positive
Fox *et al* (1998)	Yes	R	36 (14/22)	36	0	?	?	Optical	FVIII	ND	Survival curves	OS	Negative
Pavlopoulos *et al* (1998)	Yes	R	106 (56/50)	?	?	Yes	?	Automated	FVIII	Limited	Missing	Missing	Negative
Tanigawa *et al* (1997)	Yes	R	133 (76/57)	?	?	Yes	2	Optical	CD34	Advanced	Reported in text	OS	Positive
Takahashi *et al* (1997)	No	R	27 (12/15)	27	0	Yes	1	Optical	FVIII	Limited	Survival curves	RFS	Positive
Engel *et al* (1996)	No	R	35 (21/14)	?	?	Yes	2	Optical	CD31	Limited	Data extrapolated	RFS	Positive
Lindmark *et al* (1996)	No	C	212 (90/122)	124	88	Yes	1	Optical	FVIII	Limited	Data extrapolated	OS	Inverse
Mooteri *et al* (1996)	Yes	R	32 (?)	?	?	Yes	?	Optical	FVIII	Advanced	Missing	Missing	Negative
Takebayashi *et al* (1996)	Yes	R	166 (108/58)	?	?	Yes	2	Optical	FVIII	Limited	Survival curves	OS	Positive
Tomisaki *et al* (1996)	No	R	175 (98/77)	?	?	?	1	Optical	FVIII	Advanced	Data extrapolated	OS	Negative
Frank *et al* (1995)	Yes	R	105 (53/52)	105	0	Yes	?	Optical	FVIII	Limited	Missing	Missing	Positive
Bossi *et al* (1995)	Yes	R	178 (?)	?	?	?	1	Optical	CD31	Mixed	Missing	Missing	Negative
Saclarides *et al* (1994)	Yes	R	48 (33/15)	0	48	Yes	1	Optical	FVIII	Mixed	Missing	Missing	ND

C corresponds to studies including consecutive patients, R to retrospective studies without inclusion of consecutive patients. Extension means a predominance of limited forms (A/B), of advanced forms (C/D) or a balance between limited and advanced forms among the same study (mixed). RR estimate was either reported in text, or provided by mail by authors, or extrapolated from the data provided by authors in text, or estimated from the survival curves. A positive result means that there was an inverse relationship between MVD and survival, an inverse result means that there was a direct relationship between MVD and survival, and a negative result that there is no relationship. ‘Readers’ are readers of the histologic slides, ‘blinded reading’ means that readers of the slides were unaware of the clinical outcome of patients, and ‘?’ corresponds to missing data.

**Table 2:** Main characteristics of the studies relating VEGF expression to survival

**First author Year of issue (reference)**	**Study from PubMed**	**Study design**	***N* (M/F)**	**Colon (*n*)**	**Rectum (*n*)**	**Blinded reading**	**Reader(s) (*n*)**	**VEGF assessment**	**Extension**	**RR Estimate**	**Survival analysis**	**Results**
Galizia *et al* (2004)	Yes	C	104 (73/31)	104	0	Yes	2	VEGF	Limited	Reported in text	OS, RFS	Positive
Tamura *et al* (2004)	Yes	R	49 (35/14)	26	23	Yes	2	VEGF	Advanced	Reported in text	OS	Negative
Kaio *et al* (2003a, 2003b)	Yes	R	152 (94/58)	?	?	?	?	VEGF-C	Mixed	Reported in text	OS	Positive
Khorana *et al* (2003)	Yes	C	131 (69/62)	131	0	Yes	1	VEGF	Advanced	Given by authors	OS	Negative
Zheng *et al* (2003)	Yes	R	97 (58/39)	?	?	?	?	VEGF	Mixed	Reported in text	OS	Negative
Cascinu *et al* (2002)	Yes	C	79 (44/35)	0	79	Yes	2	VEGF	Advanced	Reported in text	RFS	Positive
Nanni *et al* (2002)	Yes	P	263 (137/126)	263	0	?	2	VEGF	Mixed	Data extrapolated	OS, RFS	Negative
White *et al* (2002)	Yes	R	84 (42/42)	62	22	Yes	2	VEGF-D VEGFR-3	Limited	Reported in text	OS, RFS	Positive
Cascinu *et al* (2001)	Yes	C	150 (90/60)	150	0	Yes	2	VEGF	Advanced	Data extrapolated	RFS	Positive
Harada *et al* (2001)	Yes	C	259 (?)	?	?	Yes	2	VEGF	Mixed	Survival curves	OS	Positive
Cascinu *et al* (2000)	Yes	C	121 (71/50)	121	0	Yes	2	VEGF	Limited	Data extrapolated	RFS	Positive
Lee *et al* (2000)	Yes	C	145 (80/65)	102	43	Yes	2	VEGF	Limited	Survival curves	OS	Negative
Maeda *et al* (2000)	Yes	R	100 (70/30)	?	?	Yes	2	VEGF	Mixed	Reported in text	RFS	Positive
Van Triest *et al* (2000)	Yes	R	32 (13/19)	26	6	Yes	2	VEGF	Mixed	Missing	Missing	ND
Ishigami *et al* (1998)	Yes	R	60 (40/20)	31	29	?	?	VEGF (Northern blot)	Advanced	Reported in text	OS	Positive
Tokunaga *et al* (1998)	Yes	R	61 (34/27)	61	0	?	2	VEGF isoform pattern	?	Survival curves	OS	Positive
Takahashi *et al* (1997)	Yes	R	27 (12/15)	27	0	Yes	Image analyzer	VEGF	Limited	Data extrapolated	RFS	Positive
Amaya *et al* (1997)	No	R	136 (79/57)	?	?	Yes	2	VEGF	Advanced	Reported in text	RFS	Positive

C represents studies including consecutive patients, R retrospective studies including non consecutive patients, and P prospective studies. Extension means a predominance of limited forms (A, B), of advanced forms (C, D) or a balance between the 2 forms (mixed). RR estimate was either reported in text, or provided by mail by authors, or extrapolated from the data provided by authors in text, or estimated from the survival curves. A positive result means an inverse relationship between VEGF expression and survival and a negative result means no relationship.

‘Readers’ are readers of the histologic slides, ‘blinded reading’ means that readers of the slides were unaware of the clinical outcome of patients, and ‘?’ corresponds to missing data.

## Appendix A

List of references of the twenty studies for which information was requested from the authors (Saclarides *et al*, 1994; Bossi *et al*, 1995; Frank *et al*, 1995; Mooteri *et al*, 1996; Takebayashi *et al*, 1996; Amaya *et al*, 1997; Choi *et al*, 1998; Pavlopoulos *et al*, 1998; Tokunaga *et al*, 1998; Ishikawa *et al*, 1999; Sternfeld *et al*, 1999; Lee *et al*, 2000; Pietra *et al*, 2000; van Triest *et al*, 2000; Harada *et al*, 2001; Giatromanolaki *et al*, 2002; White *et al*, 2002; Khorana *et al*, 2003; Galizia *et al*, 2004; Lackner *et al*, 2004).

## Appendix B

List of the 45 references dealing with microvessel density which were within the scope of our meta-analysis (Saclarides *et al*, 1994; Bossi *et al*, 1995; Frank *et al*, 1995; Vermeulen *et al*, 1995; Engel *et al*, 1996; Lindmark *et al*, 1996; Mooteri *et al*, 1996; Takebayashi *et al*, 1996; Tomisaki *et al*, 1996; Amaya *et al*, 1997; Takahashi *et al*, 1997; Tanigawa *et al*, 1997; Banner *et al*, 1998; Choi *et al*, 1998; Fox *et al*, 1998; Nanashima *et al*, 1998; Pavlopoulos *et al*, 1998; Abdalla *et al*, 1999; Ishikawa *et al*, 1999; Liu *et al*, 1999; Sternfeld *et al*, 1999; Vermeulen *et al*, 1999; Galindo-Gallego *et al*, 2000; Galindo Gallego *et al*, 2000; Kondo *et al*, 2000; Pietra *et al*, 2000; van Triest *et al*, 2000; Sokmen *et al*, 2001; Barozzi *et al*, 2002; Cianchi *et al*, 2002; Furudoi *et al*, 2002; Giatromanolaki *et al*, 2002; Nanni *et al*, 2002; White *et al*, 2002; Kaio *et al*, 2003a, 2003b; Li *et al*, 2003; Prall *et al*, 2003; Shan *et al*, 2003; Zheng *et al*, 2003; Galizia *et al*, 2004; Lackner *et al*, 2004; Liang *et al*, 2004; Onogawa *et al*, 2004; Saad *et al*, 2004).

## Appendix C

List of the twenty-seven references dealing with VEGF expression which were within the scope of our meta-analysis (Amaya *et al*, 1997; Takahashi *et al*, 1997; Ishigami *et al*, 1998; Nanashima *et al*, 1998; Tokunaga *et al*, 1998; Liu *et al*, 1999; Cascinu *et al*, 2000; Kondo *et al*, 2000; Lee *et al*, 2000; Maeda *et al*, 2000; Seto *et al*, 2000; van Triest *et al*, 2000; Cascinu *et al*, 2001; Harada *et al*, 2001; Barozzi *et al*, 2002; Cascinu *et al*, 2002; Furudoi *et al*, 2002; Nanni *et al*, 2002; White *et al*, 2002; Kaio *et al*, 2003a, 2003b; Khorana *et al*, 2003; Zheng *et al*, 2003; Galizia *et al*, 2004; Onogawa *et al*, 2004; Saad *et al*, 2004; Tamura *et al*, 2004).

## Appendix D

List of the twelve references using Dukes and TNM classification Takebayashi *et al*, 1996; Choi *et al*, 1998; Tokunaga *et al*, 1998; Vermeulen *et al*, 1999; Lee *et al*, 2000; Maeda *et al*, 2000; Harada *et al*, 2001; Sokmen *et al*, 2001; Cianchi *et al*, 2002; Khorana *et al*, 2003; Galizia *et al*, 2004.

## Appendix E

List of the eighteen references dealing with microvessel density and overall survival for which statistical calculation were performed (Lindmark *et al*, 1996; Takebayashi *et al*, 1996; Tomisaki *et al*, 1996; Tanigawa *et al*, 1997; Choi *et al*, 1998; Fox *et al*, 1998; Sternfeld *et al*, 1999; Vermeulen *et al*, 1999; Galindo Gallego *et al*, 2000; Sokmen *et al*, 2001; Cianchi *et al*, 2002; Nanni *et al*, 2002; Kaio *et al*, 2003b; Li *et al*, 2003; Prall *et al*, 2003; Shan *et al*, 2003; Zheng *et al*, 2003; Galizia *et al*, 2004).

## References

[bib1] Abdalla SA, Behzad F, Bsharah S, Kumar S, Amini SK, O'Dwyer ST, Haboubi NY (1999) Prognostic relevance of microvessel density in colorectal tumours. Oncol Rep 6: 839–842 10373667 10.3892/or.6.4.839

[bib2] Altman DG (2001) Systematic reviews in health care: systematic reviews of evaluations of prognostic variables. BMJ 323: 224–228 11473921 10.1136/bmj.323.7306.224 PMC1120839

[bib3] Amaya H, Tanigawa N, Lu C, Matsumura M, Shimomatsuya T, Horiuchi T, Muraoka R (1997) Association of vascular endothelial growth factor expression with tumor angiogenesis, survival and thymidine phosphorylase/platelet-derived endothelial cell growth factor expression in human colorectal cancer. Cancer Lett 119: 227–235 9570376 10.1016/s0304-3835(97)00280-2

[bib4] Banner BF, Whitehouse R, Baker SP, Swanson RS (1998) Tumor angiogenesis in stage II colorectal carcinoma: association with survival. Am J Clin Pathol 109: 733–737 9620031 10.1093/ajcp/109.6.733

[bib5] Barozzi C, Ravaioli M, D'Errico A, Grazi GL, Poggioli G, Cavrini G, Mazziotti A, Grigioni WF (2002) Relevance of biologic markers in colorectal carcinoma: a comparative study of a broad panel. Cancer 94: 647–657 11857296 10.1002/cncr.10278

[bib6] Bossi P, Viale G, Lee AK, Alfano R, Coggi G, Bosari S (1995) Angiogenesis in colorectal tumors: microvessel quantitation in adenomas and carcinomas with clinicopathological correlations. Cancer Res 55: 5049–5053 7585550

[bib7] Cascinu S, Graziano F, Catalano V, Staccioli MP, Rossi MC, Baldelli AM, Barni S, Brenna A, Secondino S, Muretto P, Catalano G (2002) An analysis of p53, BAX and vascular endothelial growth factor expression in node-positive rectal cancer. Relationships with tumour recurrence and event-free survival of patients treated with adjuvant chemoradiation. Br J Cancer 86: 744–749 11875737 10.1038/sj.bjc.6600155 PMC2375295

[bib8] Cascinu S, Graziano F, Valentini M, Catalano V, Giordani P, Staccioli MP, Rossi C, Baldelli AM, Grianti C, Muretto P, Catalano G (2001) Vascular endothelial growth factor expression, S-phase fraction and thymidylate synthase quantitation in node-positive colon cancer: relationships with tumor recurrence and resistance to adjuvant chemotherapy. Ann Oncol 12: 239–244 11300331 10.1023/a:1008339408300

[bib9] Cascinu S, Staccioli MP, Gasparini G, Giordani P, Catalano V, Ghiselli R, Rossi C, Baldelli AM, Graziano F, Saba V, Muretto P, Catalano G (2000) Expression of vascular endothelial growth factor can predict event-free survival in stage II colon cancer. Clin Cancer Res 6: 2803–2807 10914727

[bib10] Chalkley HW (1943) Method for the quantitative morphologic analysis of tissues. J Natl Cancer Inst 4: 47–53

[bib11] Choi HJ, Hyun MS, Jung GJ, Kim SS, Hong SH (1998) Tumor angiogenesis as a prognostic predictor in colorectal carcinoma with special reference to mode of metastasis and recurrence. Oncology 55: 575–581 9778626 10.1159/000011915

[bib12] Cianchi F, Palomba A, Messerini L, Boddi V, Asirelli G, Perigli G, Bechi P, Taddei A, Pucciani F, Cortesini C (2002) Tumor angiogenesis in lymph node-negative rectal cancer: correlation with clinicopathological parameters and prognosis. Ann Surg Oncol 9: 20–26 11829426 10.1245/aso.2002.9.1.20

[bib13] Cucherat M, Boissel JP, Leizorovicz A, Haugh MC (1997) EasyMA: a program for the meta-analysis of clinical trials. Comput Methods Programs Biomed 53: 187–190 9230453 10.1016/s0169-2607(97)00016-3

[bib14] Engel CJ, Bennett ST, Chambers AF, Doig GS, Kerkvliet N, O'Malley FP (1996) Tumor angiogenesis predicts recurrence in invasive colorectal cancer when controlled for Dukes staging. Am J Surg Pathol 20: 1260–1265 8827033 10.1097/00000478-199610000-00012

[bib15] Fox SH, Whalen GF, Sanders MM, Burleson JA, Jennings K, Kurtzman S, Kreutzer D (1998) Angiogenesis in normal tissue adjacent to colon cancer. J Surg Oncol 69: 230–234 9881940 10.1002/(sici)1096-9098(199812)69:4<230::aid-jso7>3.0.co;2-q

[bib16] Frank RE, Saclarides TJ, Leurgans S, Speziale NJ, Drab EA, Rubin DB (1995) Tumor angiogenesis as a predictor of recurrence and survival in patients with node-negative colon cancer. Ann Surg 222: 695–699 8526575 10.1097/00000658-199512000-00002 PMC1235017

[bib17] Furudoi A, Tanaka S, Haruma K, Kitadai Y, Yoshihara M, Chayama K, Shimamoto F (2002) Clinical significance of vascular endothelial growth factor C expression and angiogenesis at the deepest invasive site of advanced colorectal carcinoma. Oncology 62: 157–166 11914602 10.1159/000048262

[bib18] Galindo-Gallego M, Fernandez-Acenero MJ, Sanz-Ortega J, Aljama A, Lopez-Elzaurdia C (2000) Prognostic significance of microvascular counts in rectal carcinoma. Pathol Res Pract 196: 607–612 10997734 10.1016/S0344-0338(00)80002-3

[bib19] Galindo Gallego M, Fernandez Acenero MJ, Sanz Ortega J, Aljama A (2000) Vascular enumeration as a prognosticator for colorectal carcinoma. Eur J Cancer 36: 55–60 10741295 10.1016/s0959-8049(99)00243-9

[bib20] Galizia G, Lieto E, Ferraraccio F, Orditura M, De Vita F, Castellano P, Imperatore V, Romano C, Ciardiello F, Agostini B, Pignatelli C (2004) Determination of molecular marker expression can predict clinical outcome in colon carcinomas. Clin Cancer Res 10: 3490–3499 15161706 10.1158/1078-0432.CCR-0960-03

[bib21] Garcea G, Lloyd TD, Gescher A, Dennison AR, Steward WP, Berry DP (2004) Angiogenesis of gastrointestinal tumours and their metastases--a target for intervention? Eur J Cancer 40: 1302–1313 15177488 10.1016/j.ejca.2004.02.015

[bib22] Giatromanolaki A, Sivridis E, Minopoulos G, Polychronidis A, Manolas C, Simopoulos C, Koukourakis MI (2002) Differential assessment of vascular survival ability and tumor angiogenic activity in colorectal cancer. Clin Cancer Res 8: 1185–1191 12006536

[bib23] Gill S, Loprinzi CL, Sargent DJ, Thome SD, Alberts SR, Haller DG, Benedetti J, Francini G, Shepherd LE, Francois Seitz J, Labianca R, Chen W, Cha SS, Heldebrant MP, Goldberg RM (2004) Pooled analysis of fluorouracil-based adjuvant therapy for stage II and III colon cancer: who benefits and by how much? J Clin Oncol 22: 1797–1806 15067028 10.1200/JCO.2004.09.059

[bib24] Harada Y, Ogata Y, Shirouzu K (2001) Expression of vascular endothelial growth factor and its receptor KDR (kinase domain-containing receptor)/Flk-1 (fetal liver kinase-1) as prognostic factors in human colorectal cancer. Int J Clin Oncol 6: 221–228 11723743 10.1007/pl00012109

[bib25] Hayes DF, Isaacs C, Stearns V (2001) Prognostic factors in breast cancer: current and new predictors of metastasis. J Mammary Gland Biol Neoplasia 6: 375–392 12013528 10.1023/a:1014778713034

[bib26] Hellman S, Rosenberg SA (2001) Cancers of the gastrointestinal tract. In Cancer: principles and practice of oncology Vincent Jr TD (ed) 6th ed, pp 1230–1238. Philadelphia, PA: Lippincott

[bib27] Hermanek P, Sobin LH (1995) Prognostic factors in colorectal cancer. In Prognostic factors in cancer Hermanek P (ed) pp 64–79. Berlin: Springer

[bib28] Hurwitz H, Fehrenbacher L, Novotny W, Cartwright T, Hainsworth J, Heim W, Berlin J, Baron A, Griffing S, Holmgren E, Ferrara N, Fyfe G, Rogers B, Ross R, Kabbinavar F (2004) Bevacizumab plus irinotecan, fluorouracil, and leucovorin for metastatic colorectal cancer. N Engl J Med 350: 2335–2342 15175435 10.1056/NEJMoa032691

[bib29] Ishigami SI, Arii S, Furutani M, Niwano M, Harada T, Mizumoto M, Mori A, Onodera H, Imamura M (1998) Predictive value of vascular endothelial growth factor (VEGF) in metastasis and prognosis of human colorectal cancer. Br J Cancer 78: 1379–1384 9823983 10.1038/bjc.1998.688 PMC2063176

[bib30] Ishikawa H, Fujii H, Yamamoto K, Morita T, Hata M, Koyama F, Terauchi S, Sugimori S, Kobayashi T, Enomoto H, Yoshikawa S, Nishikawa T, Nakano H (1999) Tumor angiogenesis predicts recurrence with normal serum carcinoembryonic antigen in advanced rectal carcinoma patients. Surg Today 29: 983–991 10554319 10.1007/s005950050633

[bib31] Jubb AM, Hurwitz HI, Bai W, Holmgren EB, Tobin P, Guerrero AS, Kabbinavar F, Holden SN, Novotny WF, Frantz GD, Hillan KJ, Koeppen H (2006) Impact of vascular endothelial growth factor-A expression, thrombospondin-2 expression, and microvessel density on the treatment effect of bevacizumab in metastatic colorectal cancer. J Clin Oncol 24: 217–227 16365183 10.1200/JCO.2005.01.5388

[bib32] Kaio E, Tanaka S, Kitadai Y, Sumii M, Yoshihara M, Haruma K, Chayama K (2003a) Clinical significance of angiogenic factor expression at the deepest invasive site of advanced colorectal carcinoma. Oncology 64: 61–73 12457033 10.1159/000066511

[bib33] Kaio E, Tanaka S, Oka S, Hiyama T, Kitadai Y, Haruma K, Chayama K (2003b) Clinical significance of thrombospondin-1 expression in relation to vascular endothelial growth factor and interleukin-10 expression at the deepest invasive tumor site of advanced colorectal carcinoma. Int J Oncol 23: 901–911 12963968

[bib34] Khorana AA, Ryan CK, Cox C, Eberly S, Sahasrabudhe DM (2003) Vascular endothelial growth factor, CD68, and epidermal growth factor receptor expression and survival in patients with Stage II and Stage III colon carcinoma: a role for the host response in prognosis. Cancer 97: 960–968 12569594 10.1002/cncr.11152

[bib35] Kondo Y, Arii S, Furutani M, Isigami S, Mori A, Onodera H, Chiba T, Imamura M (2000) Implication of vascular endothelial growth factor and p53 status for angiogenesis in noninvasive colorectal carcinoma. Cancer 88: 1820–1827 10760758

[bib36] Lackner C, Jukic Z, Tsybrovskyy O, Jatzko G, Wette V, Hoefler G, Klimpfinger M, Denk H, Zatloukal K (2004) Prognostic relevance of tumour-associated macrophages and von Willebrand factor-positive microvessels in colorectal cancer. Virchows Arch 445: 160–167 15232739 10.1007/s00428-004-1051-z

[bib37] Lee JC, Chow NH, Wang ST, Huang SM (2000) Prognostic value of vascular endothelial growth factor expression in colorectal cancer patients. Eur J Cancer 36: 748–753 10762747 10.1016/s0959-8049(00)00003-4

[bib38] Li C, Gardy R, Seon BK, Duff SE, Abdalla S, Renehan A, O'Dwyer ST, Haboubi N, Kumar S (2003) Both high intratumoral microvessel density determined using CD105 antibody and elevated plasma levels of CD105 in colorectal cancer patients correlate with poor prognosis. Br J Cancer 88: 1424–1431 12778073 10.1038/sj.bjc.6600874 PMC2741032

[bib39] Liang JT, Huang KC, Jeng YM, Lee PH, Lai HS, Hsu HC (2004) Microvessel density, cyclo-oxygenase 2 expression, K-ras mutation and p53 overexpression in colonic cancer. Br J Surg 91: 355–361 14991639 10.1002/bjs.4447

[bib40] Lindmark G, Gerdin B, Sundberg C, Pahlman L, Bergstrom R, Glimelius B (1996) Prognostic significance of the microvascular count in colorectal cancer. J Clin Oncol 14: 461–466 8636758 10.1200/JCO.1996.14.2.461

[bib41] Liu X, Song S, Xu W (1999) Microvessel quantitation and expression of VEGF in colorectal tumors. Zhonghua Zhong Liu Za Zhi 21: 430–432 11776618

[bib42] Maeda K, Nishiguchi Y, Yashiro M, Yamada S, Onoda N, Sawada T, Kang SM, Hirakawa K (2000) Expression of vascular endothelial growth factor and thrombospondin-1 in colorectal carcinoma. Int J Mol Med 5: 373–378 10719053 10.3892/ijmm.5.4.373

[bib43] Mamounas E, Wieand S, Wolmark N, Bear HD, Atkins JN, Song K, Jones J, Rockette H (1999) Comparative efficacy of adjuvant chemotherapy in patients with Dukes' B *versus* Dukes' C colon cancer: results from four National Surgical Adjuvant Breast and Bowel Project adjuvant studies (C-01, C-02, C-03, and C-04). J Clin Oncol 17: 1349–1355 10334518 10.1200/JCO.1999.17.5.1349

[bib44] Mooteri S, Rubin D, Leurgans S, Jakate S, Drab E, Saclarides T (1996) Tumor angiogenesis in primary and metastatic colorectal cancers. Dis Colon Rectum 39: 1073–1080 8831518 10.1007/BF02081403

[bib45] Nanashima A, Ito M, Sekine I, Naito S, Yamaguchi H, Nakagoe T, Ayabe H (1998) Significance of angiogenic factors in liver metastatic tumors originating from colorectal cancers. Dig Dis Sci 43: 2634–2640 9881494 10.1023/a:1026643009152

[bib46] Nanni O, Volpi A, Frassineti GL, De Paola F, Granato AM, Dubini A, Zoli W, Scarpi E, Turci D, Oliverio G, Gambi A, Amadori D (2002) Role of biological markers in the clinical outcome of colon cancer. Br J Cancer 87: 868–875 12373601 10.1038/sj.bjc.6600569 PMC2376168

[bib47] Onogawa S, Kitadai Y, Tanaka S, Kuwai T, Kimura S, Chayama K (2004) Expression of VEGF-C and VEGF-D at the invasive edge correlates with lymph node metastasis and prognosis of patients with colorectal carcinoma. Cancer Sci 95: 32–39 14720324 10.1111/j.1349-7006.2004.tb03167.x PMC11159672

[bib48] Papamichael D (2001) Prognostic role of angiogenesis in colorectal cancer. Anticancer Res 21: 4349–4353 11908690

[bib49] Parmar MK, Torri V, Stewart L (1998) Extracting summary statistics to perform meta-analyses of the published literature for survival endpoints. Stat Med 17: 2815–2834 9921604 10.1002/(sici)1097-0258(19981230)17:24<2815::aid-sim110>3.0.co;2-8

[bib50] Pavlopoulos PM, Konstantinidou AE, Agapitos E, Kavantzas N, Nikolopoulou P, Davaris P (1998) A morphometric study of neovascularization in colorectal carcinoma. Cancer 83: 2067–2075 9827710

[bib51] Pietra N, Sarli L, Caruana P, Cabras A, Costi R, Gobbi S, Bordi C, Peracchia A (2000) Is tumour angiogenesis a prognostic factor in patients with colorectal cancer and no involved nodes? Eur J Surg 166: 552–556 10965834 10.1080/110241500750008628

[bib52] Poon RT, Fan ST, Wong J (2003) Clinical significance of angiogenesis in gastrointestinal cancers: a target for novel prognostic and therapeutic approaches. Ann Surg 238: 9–28 12832961 10.1097/01.sla.0000075047.47175.35 PMC1422670

[bib53] Prall F, Gringmuth U, Nizze H, Barten M (2003) Microvessel densities and microvascular architecture in colorectal carcinomas and their liver metastases: significant correlation of high microvessel densities with better survival. Histopathology 42: 482–491 12713626 10.1046/j.1365-2559.2003.01610.x

[bib54] Saad RS, Liu YL, Nathan G, Celebrezze J, Medich D, Silverman JF (2004) Endoglin (CD105) and vascular endothelial growth factor as prognostic markers in colorectal cancer. Mod Pathol 17: 197–203 14657950 10.1038/modpathol.3800034

[bib55] Saclarides TJ, Speziale NJ, Drab E, Szeluga DJ, Rubin DB (1994) Tumor angiogenesis and rectal carcinoma. Dis Colon Rectum 37: 921–926 7521279 10.1007/BF02052599

[bib56] Seto S, Onodera H, Kaido T, Yoshikawa A, Ishigami S, Arii S, Imamura M (2000) Tissue factor expression in human colorectal carcinoma: correlation with hepatic metastasis and impact on prognosis. Cancer 88: 295–301 10640960 10.1002/(sici)1097-0142(20000115)88:2<295::aid-cncr8>3.0.co;2-u

[bib57] Shan YS, Lee JC, Chow NH, Yang HB, Wang ST (2003) Immunohistochemical microvessel count is not a reliable prognostic predictor in colorectal carcinoma. Hepatogastroenterology 50: 1316–1320 14571726

[bib58] Sokmen S, Sarioglu S, Fuzun M, Terzi C, Kupelioglu A, Aslan B (2001) Prognostic significance of angiogenesis in rectal cancer: a morphometric investigation. Anticancer Res 21: 4341–4348 11908689

[bib59] Sternfeld T, Foss HD, Kruschewski M, Runkel N (1999) The prognostic significance of tumor vascularization in patients with localized colorectal cancer. Int J Colorectal Dis 14: 272–276 10663893 10.1007/s003840050227

[bib60] Stroup DF, Berlin JA, Morton SC, Olkin I, Williamson GD, Rennie D, Moher D, Becker BJ, Sipe TA, Thacker SB (2000) Meta-analysis of observational studies in epidemiology: a proposal for reporting. Meta-analysis Of Observational Studies in Epidemiology (MOOSE) group. JAMA 283: 2008–2012 10789670 10.1001/jama.283.15.2008

[bib61] Takahashi Y, Tucker SL, Kitadai Y, Koura AN, Bucana CD, Cleary KR, Ellis LM (1997) Vessel counts and expression of vascular endothelial growth factor as prognostic factors in node-negative colon cancer. Arch Surg 132: 541–546 9161399 10.1001/archsurg.1997.01430290087018

[bib62] Takebayashi Y, Aklyama S, Yamada K, Akiba S, Aikou T (1996) Angiogenesis as an unfavorable prognostic factor in human colorectal carcinoma. Cancer 78: 226–231 8673996 10.1002/(SICI)1097-0142(19960715)78:2<226::AID-CNCR6>3.0.CO;2-J

[bib63] Tamura M, Oda M, Tsunezuka Y, Matsumoto I, Kawakami K, Watanabe G (2004) Vascular endothelial growth factor expression in metastatic pulmonary tumor from colorectal carcinoma: utility as a prognostic factor. J Thorac Cardiovasc Surg 128: 517–522 15457151 10.1016/j.jtcvs.2004.03.056

[bib64] Tanigawa N, Amaya H, Matsumura M, Lu C, Kitaoka A, Matsuyama K, Muraoka R (1997) Tumor angiogenesis and mode of metastasis in patients with colorectal cancer. Cancer Res 57: 1043–1046 9067267

[bib65] Tokunaga T, Oshika Y, Abe Y, Ozeki Y, Sadahiro S, Kijima H, Tsuchida T, Yamazaki H, Ueyama Y, Tamaoki N, Nakamura M (1998) Vascular endothelial growth factor (VEGF) mRNA isoform expression pattern is correlated with liver metastasis and poor prognosis in colon cancer. Br J Cancer 77: 998–1002 9528847 10.1038/bjc.1998.164 PMC2150098

[bib66] Tomisaki S, Ohno S, Ichiyoshi Y, Kuwano H, Maehara Y, Sugimachi K (1996) Microvessel quantification and its possible relation with liver metastasis in colorectal cancer. Cancer 77: 1722–1728 8608569 10.1002/(SICI)1097-0142(19960415)77:8<1722::AID-CNCR46>3.0.CO;2-Z

[bib67] Uzzan B, Nicolas P, Cucherat M, Perret GY (2004) Microvessel density as a prognostic factor in women with breast cancer: a systematic review of the literature and meta-analysis. Cancer Res 64: 2941–2955 15126324 10.1158/0008-5472.can-03-1957

[bib68] van Triest B, Pinedo HM, Blaauwgeers JL, van Diest PJ, Schoenmakers PS, Voorn DA, Smid K, Hoekman K, Hoitsma HF, Peters GJ (2000) Prognostic role of thymidylate synthase, thymidine phosphorylase/platelet-derived endothelial cell growth factor, and proliferation markers in colorectal cancer. Clin Cancer Res 6: 1063–1072 10741735

[bib69] Vermeulen PB, Gasparini G, Fox SB, Colpaert C, Marson LP, Gion M, Belien JA, de Waal RM, Van Marck E, Magnani E, Weidner N, Harris AL, Dirix LY (2002) Second international consensus on the methodology and criteria of evaluation of angiogenesis quantification in solid human tumours. Eur J Cancer 38: 1564–1579 12142044 10.1016/s0959-8049(02)00094-1

[bib70] Vermeulen PB, Van den Eynden GG, Huget P, Goovaerts G, Weyler J, Lardon F, Van Marck E, Hubens G, Dirix LY (1999) Prospective study of intratumoral microvessel density, p53 expression and survival in colorectal cancer. Br J Cancer 79: 316–322 9888475 10.1038/sj.bjc.6690051 PMC2362196

[bib71] Vermeulen PB, Verhoeven D, Fierens H, Hubens G, Goovaerts G, Van Marck E, De Bruijn EA, Van Oosterom AT, Dirix LY (1995) Microvessel quantification in primary colorectal carcinoma: an immunohistochemical study. Br J Cancer 71: 340–343 7530985 10.1038/bjc.1995.68 PMC2033605

[bib72] Wali RK, Roy HK, Kim YL, Liu Y, Koetsier JL, Kunte DP, Goldberg MJ, Turzhitsky V, Backman V (2005) Increased microvascular blood content is an early event in colon carcinogenesis. Gut 54: 654–660 15831911 10.1136/gut.2004.056010 PMC1262671

[bib73] Weidner N, Semple JP, Welch WR, Folkman J (1991) Tumor angiogenesis and metastasis--correlation in invasive breast carcinoma. N Engl J Med 324: 1–8 10.1056/NEJM199101033240101 1701519

[bib74] Weitz J, Koch M, Debus J, Hohler T, Galle PR, Buchler MW (2005) Colorectal cancer. Lancet 365: 153–165 15639298 10.1016/S0140-6736(05)17706-X

[bib75] White JD, Hewett PW, Kosuge D, McCulloch T, Enholm BC, Carmichael J, Murray JC (2002) Vascular endothelial growth factor-D expression is an independent prognostic marker for survival in colorectal carcinoma. Cancer Res 62: 1669–1675 11912138

[bib76] Zheng S, Han MY, Xiao ZX, Peng JP, Dong Q (2003) Clinical significance of vascular endothelial growth factor expression and neovascularization in colorectal carcinoma. World J Gastroenterol 9: 1227–1230 12800229 10.3748/wjg.v9.i6.1227 PMC4611789

[bib77] Zlobec I, Steele R, Compton CC (2005) VEGF as a predictive marker of rectal tumor response to preoperative radiotherapy. Cancer 104: 2517–2521 16222693 10.1002/cncr.21484

